# Child in India

**DOI:** 10.4103/0019-5545.42393

**Published:** 2008

**Authors:** Shastri Priyavadan Chandrakant

**Affiliations:** Dr. Shastri's Clinic, Bldg. No. 3, Block No. 3, Vivina Cooperative Housing Society, S. V. Road, Andheri (West), Mumbai - 400058, India

“National Human Rights Commission will guard right to education and health.” Focus will be on life, survival, health, and basic education. Elementary education and primary health services in rural areas will get top priority.

S. Rajendra Babu,

Former Chief Justice of India, Chairperson of NHRC

September 2007

Dr. R. Srinivasmurthy (1993) also noted the clinical preoccupation of the available mental health professionals of the country and the delay of these professionals to spearhead work toward rectifying this major lacuna in liaison with the sectors like welfare, education, labor, and health along with law over the years.

India presents a unique case in terms of the sheer size of its population and 46% of them are children; characterized by heterogeneity in respect of physical, economical, social, and cultural conditions. Its population of 1.12 billion constitutes 16% of the world population, with 74% of them living in rural areas.

India is a secular state with various languages, cultures, and religions. It has 31 states, 1618 languages, 544 dialects, and 1942 mother tongues with 148 mediums of instruction at school level. India publishes more than 27,000 daily newspapers and periodicals covering the range of languages and cultural diversity unparallel to any other country in the world. A total of 6400 castes and six religions make universal acceptance of any program difficult. This kind of complex and multifaceted country makes formulation of National policies, programming, and planning quite a challenging task. Each and every one of the 600 districts of India is unique in many ways. Each district will need its planning at local level. For such a diversified country, it is difficult to envisage a national program that fits all and even of all are considered in reality it may fit none.

The constitution of India envisages the establishment of new social order based on equality, freedom, justice, and the dignity of the individual. It aims at the elimination of poverty, ignorance, and ill health, and directs the state with regard to raising the level of nutrition and of the people; securing the health and specially ensuring that children are given opportunity to develop in a healthy manner.

India has been a signatory to all the resolutions including the latest passed on January 1, 1996 which states that every child will have equal opportunities, protection of rights, and full participation (The Person with Disability Act 1995). After six decades of independence, we have managed to resolve to help the Indian child. Child has never been given even minimum attention and essential requirements in last six decades. It is not surprising that under the “minimum need programs,” in last 10 years, outlays and expenditure under health sector though very small, are never spent.

India is a home to almost 19% of the world' s children. More than one-third of the country' s population, around 480 million, is below 18 years. A total of 560 millions are below the age of 25 years (54% of the population). According to one assumption, 40% of these children are in need of care and protection, which indicates the extent of the problem. In a country like India with its multicultural, multiethnic, and multireligious population, the problems of socially marginalized and economically backward groups are immense. Within such groups, the most vulnerable section is always the children. For the Ministry of Women and Child Development, the challenge is to reach out to the most vulnerable and socially excluded child of this country and create an environment wherein, not only is every child protected, but also has access to opportunities and education for all round growth and development.

Growth alone does not deliver people out of poverty. Government must focus on the single biggest cause of vulnerability in health spending. A concerted focus on public health can change the lives of millions. National Commission for Enterprise in Unorganized Sector (NCEUS) reports 77% of the population living below the poverty line (Rupees 20 per capita).

Independent India has taken large strides in addressing issues like child education, health, and development. But, it has failed to implement program which is progressive, promotional, performance based, preventive, and protective to the child. However, child protection has remained largely unaddressed. There is now a realization that if issues of child abuse and neglect like female foeticide and infanticide, girl child discrimination, child marriage, trafficking of children, and so on are not addressed, it will affect the overall progress of the country.

Traditionally in India, the responsibility of care and protection of children has been with families and communities. A strong knit patriarchal family that is meant to look after its children well has seldom had the realization that children are individuals with their own rights. While the Constitution of India guarantees many fundamental rights to the children, the approach to ensure the fulfilment of these rights was always more need based rather than rights based. The transition to the rights-based approach in the Government and civil society is still evolving.

It has very clearly emerged that across different kinds of abuse, it is young children, in the 5-12 year group, who are most at risk of abuse and exploitation. There is an enormous number of children that the country has to take care of. While articulating its vision of progress, development, and equity, India has expressed its recognition of the fact that when its children are educated, healthy, happy, and have access to opportunities, they are the country' s greatest human resource.

**Critical Concerns:**

- Every fifth child in the world lives in India.
- Every third malnourished child in the world lives in India.
- Every second Indian child is underweight.
- Three out of four children in India are anemic.
- Every second new born has reduced learning capacity due to iodine deficiency.
- Decline in female/male ratio is maximum in 0-6 years: 927 females per 1000 males.
- Birth registration is just 62% (Registrar General of India [RGI]-2004).
- Retention rate at primary level is 71.01% (Elementary education in India progress toward UEE NUEPA Flash Statistics DISE 2005-2006).
- Girls' enrolment in schools at primary level is 47.79% (Elementary education in India progress toward UEE NUEPA Flash Statistics DISE 2005-2006).
- A total of 1104 lakh child labor in the country (SRO 2000).
- IMR is as high as 58 per 1000 live births (SRS 2005).
- MMR is equally high at 301 per 100,000 live births (SRS 2001-2003).
- Children born with low birth weight are 46% (NFHS-III).
- Children under 3 with anemia are 79% (NFHS-III).
- Immunization coverage is very low (polio-78.2%, measles - 58.8%, DPT - 55.3%, BCG - 78% (NFHS-III)).

Examining the government policies and national program for promoting child mental health, it becomes evident that there is a wide gap between the children' s needs and existing resources. There is neither an independent nor integrated child mental health policy in India. The multiple needs of a child are currently covered by different policies and subsequently different ministries. It is crucial to develop a comprehensive policy to cover all aspects of children' s mental health, under one umbrella.

The incidence of children needing mental health services is high. Even after 61 years of independence, resources to meet the mental health needs of children, human power, as well as preventive, diagnostic, and treatment services, are extremely limited. What is the gap due to? Inadequate government policy, unaroused citizenry, insufficient resources, or the lackadaisical attitude of people toward the needs of children.

In spite of multiple legal provisions to provide child all the rights and privileges, Indian child continues to struggle and face challenges in the form of deficiencies and deprivations.

Single window operation for child mental health, education, and welfare will surely go a long way in successful implementation of various plans and policies related to child in India.

